# Assessment of Noise of MEMS IMU Sensors of Different Grades for GNSS/IMU Navigation

**DOI:** 10.3390/s24061953

**Published:** 2024-03-19

**Authors:** Vladimir Suvorkin, Miquel Garcia-Fernandez, Guillermo González-Casado, Mowen Li, Adria Rovira-Garcia

**Affiliations:** 1 Rokubun S.L., Paral·lel 88-1, 08015 Barcelona, Spain; miquel.garcia@rokubun.cat; 2 Research Group of Astronomy and Geomatics (gAGE), Universitat Politècnica de Catalunya (UPC), Campus Nord, Jordi Girona 1-3, 08034 Barcelona, Spain; guillermo.gonzalez@upc.edu (G.G.-C.); mowen.li@upc.edu (M.L.); adria.rovira@upc.edu (A.R.-G.); 3 Institute of Space Sciences, Shandong University, Weihai 264209, China

**Keywords:** IMU, MEMS, OADEV, GNSS, sensor fusion, loosely coupled

## Abstract

Inertial measurement units (IMUs) are key components of various applications including navigation, robotics, aerospace, and automotive systems. IMU sensor characteristics have a significant impact on the accuracy and reliability of these applications. In particular, noise characteristics and bias stability are critical for proper filter settings to perform a combined GNSS/IMU solution. This paper presents an analysis based on the Allan deviation of different IMU sensors that correspond to different grades of micro-electromechanical systems (MEMS)-type IMUs in order to evaluate their accuracy and stability over time. The study covers three IMU sensors of different grades (ascending order): Rokubun Argonaut navigator sensor (InvenSense TDK MPU9250), Samsung Galaxy Note10 phone sensor (STMicroelectronics LSM6DSR), and NovAtel PwrPak7 sensor (Epson EG320N). The noise components of the sensors are computed using overlapped Allan deviation analysis on data collected over the course of a week in a static position. The focus of the analysis is to characterize the random walk noise and bias stability, which are the most critical for combined GNSS/IMU navigation and may differ or may not be listed in manufacturers’ specifications. Noise characteristics are calculated for the studied sensors and examples of their use in loosely coupled GNSS/IMU processing are assessed. This work proposes a structured and reproducible approach for working with sensors for their use in navigation tasks in combination with GNSS, and can be used for sensors of different levels to supplement missing or incorrect sensor manufacturers’ data.

## 1. Introduction

Micro-electromechanical system (MEMS) sensors have emerged as indispensable components in a wide array of modern technological applications. These sensors, often integrated onto a single chip, can measure acceleration, angular rate, and magnetic field strength.

MEMS sensors are widespread because of several factors [1]: first and foremost, their miniature size allows them to be seamlessly integrated into compact electronic devices, allowing for the creation of devices that are both portable and powerful; furthermore, MEMS sensors are cost effective to produce in large quantities, making them economically viable for mass-market applications; their solid-state design, with no moving parts, ensures durability and resistance to shock and vibration, making them suitable for harsh operating conditions.

Triaxial inertial measurement units (IMUs) integrate MEMS of different types as strapdown systems [2] with accelerometers and gyroscopes. They are used in determining an object’s orientation, acceleration, and velocity for navigational tasks. Therefore, the applications of IMUs include aircraft, autonomous vehicles, robotics, and even consumer-grade Global Navigation Satellite System (GNSS) devices, enhancing their performance by providing continuous position and orientation information when GNSS signals are temporarily unavailable, such as in tunnels or urban canyons.

Due to the noise of IMU sensor measurements and the presence of drifting biases in the measurements, it is not possible to perform inertial-only navigation with sufficient accuracy. This is usually palliated by using additional systems such as GNSS, which allow for estimation of the bias values. In this case, GNSS and sensor readings are combined to smooth the position estimates and to increase position availability, mitigating conditions where GNSS is not available at characteristic time intervals, when the accuracy degradation remains within acceptable limits [3]. This degradation depends on IMU noise, bias stability, and how well the bias values were determined before the GNSS signal was lost. In turn, the latter critically depends on the correct navigation filter settings, which are directly related to the noise characteristics of IMUs, specifically on angular (for gyroscopes) and velocity (for accelerometers) random walk and bias stability.

High-grade MEMS sensors feature lower noise levels and higher bias stability, allowing for more accurate and reliable navigation solutions. Thus, understanding these noise parameters is essential for designing effective sensor fusion algorithms and filters to mitigate errors and improve overall system performance.

In practice, the actual performance of MEMS sensors may deviate from the specifications provided by manufacturers [4]. Environmental conditions, temperature variations, and manufacturing tolerances can all contribute to these discrepancies. In addition, the actual values may be different in case of operation at other sampling rates than those for which the parameters were measured by the manufacturer. In some cases, manufacturers do not provide data at all for the required noise components. Therefore, thorough calibration and characterization of MEMS sensors are often necessary to ensure their accuracy in specific applications. One must carefully assess the noise characteristics of MEMS sensors to achieve the desired level of accuracy and reliability [5] or at least use them as a starting point for further small changes. The use of sensors in autonomous inertial navigation systems (INS) requires thorough testing in the laboratory under static and dynamic conditions [6]. But for the purpose of use in the combined GNSS/IMU mode, the proposed procedure for evaluating noise components under static conditions seems to be sufficient.

This task can be performed in advance using the Allan variation method, which initially was applied for characterizing the stability of atomic clocks [7]. Later, it was widely used for IMU analysis [8,9] and even adopted as an IEEE standard, though just for fiber optic and laser gyros [10,11]. More specifically, this method is called the Allan variance slope method because the slopes on the Allan plot refer to different components of the total noise. This method, however, has been criticized [12] because it does not allow for identification of some weaker components of the noise and, therefore, more accurate methods are being developed [13,14]. Nevertheless, in the present study, the focus is placed on two main components, noise random walk and bias stability, which are usually used for error-state extended Kalman filter implementations of GNSS/IMU sensor fusion [15]. For these components, the classical method is sufficient, and therefore, has been adopted as the method for the present analysis.

## 2. Equipment and Experimental Setup

Three devices with embedded MEMS IMUs of different grades were used to collect accelerometer and gyroscope measurements:

1. Geodetic class GNSS receiver NovAtel PwrPak7 with sensor EPSON G320N [16]; manufacturer: NovAtel Inc., Calgary, AB, Canada. The device is owned by the UPC.
2. Smartphone Samsung Galaxy Note10 with sensor STMicroelectronics LSM6DSR  [17]; manufacturer: Samsung Electronics Co., Ltd., Suwon, Republic of Korea. The device is owned by Rokubun S.L.
3. Mass-market Rokubun Argonaut GNSS receiver with sensor InvenSense TDK MPU9250 [18]; manufacturer Rokubun S.L., Barcelona, Spain. The device is owned by Rokubun S.L.

The key specifications of these sensors are summarized in Table 1.

**Figure 1:**
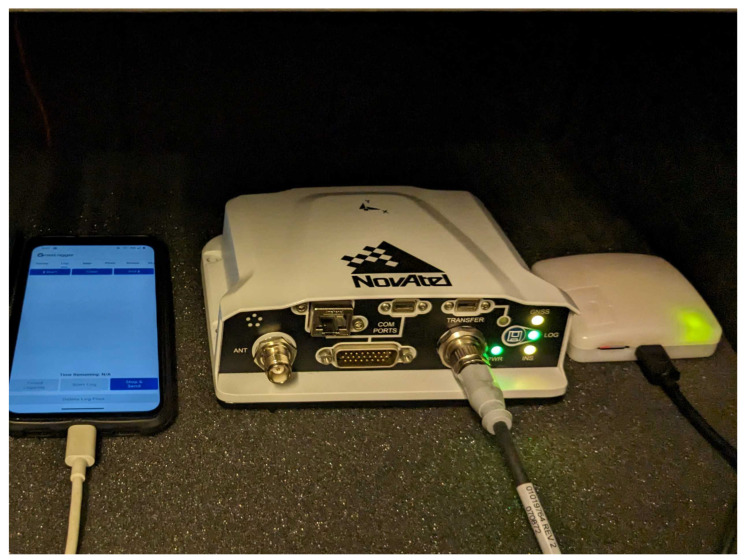
Data collecting experiment setup. From left to right: Samsung Galaxy Note10, NovAtel PwrPak7, Rokubun Argonaut.

The data acquisition was performed in an isolated room with relatively stable temperature, and the devices were placed on foam pads to minimize vibrations in the supporting structures as it shown on the Figure 1. Data collection was carried out over a period of 7 days. The z-axes of the sensors were in the vertical direction, and the x- and y-axes were in the horizontal plane and roughly co-aligned for all the devices involved in the experiment.

To collect the EPSON G320N’s measurements, the receiver NovAtel PwrPak7 IMU was configured to record only raw IMU measurements with consecutive binary logs of 4 h each. The IMU measurements were then extracted from these logs and written as CSV files using a custom Python script, interpreted in Python version 3.9.13.

The TDK MPU9250’s IMU measurements were recorded in Argonaut receiver in a binary format, the data from which were converted to text in tabular form using Rokubun proprietary software rokubun_core version 75.3.1.

Collecting data on a smartphone over a long period of time has certain limitations, attributable to the fact that these devices are not generally designed for such tasks. Out of a number of smartphones initially available for testing in this study—Xiaomi Mi 8 (manufacturer Xiaomi Corp., Beijing, China), Google Pixel 4 (manufacturer: Google LLC, Mountain View, CA, USA) and Samsung Galaxy Note10—only with the latter was it possible to record the necessary data. The apps tried were phyphox version 1.1.12 [19], Sensor Logger version 1.18 [20], and GNSS Logger version 3.0.6.3 [21].

Sensor Logger failed to run on the Google Pixel 4. The *Sensor Logger* app on other smartphones showed instability—at some point the app stopped working. Similar problems occurred with the phyphox application with intervals larger than one day: the application also terminated or rebooted before the end of the timer countdown. These problems are probably related both to the peculiarities of the operation of the logger programs themselves, which do not write data to internal storage continuously, but keep them in the random access memory, and to the operation of the Android operating system, which for some reason may clear memory or restart the application. The GNSS Logger app worked on the Samsung Galaxy Note10 for the whole week without any problems. The application’s internal timer cannot be set for more than 24 h, but it can perform uninterrupted data collection for a reasonably unlimited amount of time, which is started and stopped manually by pressing the corresponding buttons, so it was necessary to truncate the data at the beginning and the end of the time series.

## 3. Methodology

To analyze the noise characteristics of the sensors, the so-called Allan deviation (ADEV) slope method was used. The ADEV analysis method, originally formulated by David W. Allan in the 1960s [7], has evolved with the introduction of ADEV slopes, enhancing its capability to provide insights into IMU sensor noise characteristics across various time scales. The Overlapped Allan Deviation (OADEV) analysis method offers a refined approach to understanding the noise and stability characteristics of IMU sensors. The OADEV is an extension of the Allan Deviation method, aiming to account for temporal overlap or redundancy in the sensor data. For a time series of sensor measurements, the OADEV at an averaging time τ is defined as

(1) $$\sigma^{2}\left(m\tau_{0}\right)=\frac{\sum_{n=1}^{N-2m}{\left(x_{n+2m}-2x_{n+1m}+x_{n}\right)}^{2}}{2{\left(m\tau_{0}\right)}^{2}\left(N-2m\right)},$$

where $\tau =\tau_{0}m$ represents the averaging time, *m* is the averaging factor, and $x_{n}$ is the time series of measurements spaced by the basic interval $\tau_{0}$ with length *N*.

**Figure 2:**
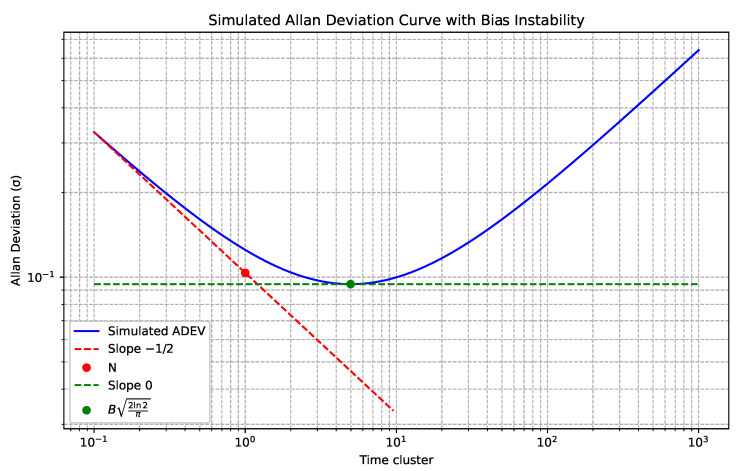
Noise random walk and bias stability on a simulated ADEV plot.

The slope method is used to interpret the Allan deviation curve on a logarithmic scale. Different components of the curve correspond to different constituents of the total sensor noise. How this looks on the plot is shown in the example of simulated ADEV data in the Figure 2. To simulate the data, time series of values were generated with added noise and bias. The components needed to combine GNSS and IMU-based inertial navigation are:

- The line with slope equal to −1/2, which yields *N*, representing an angle random walk for gyroscopes and a velocity random walk for accelerometers;
- The line with slope equal to 0, which yields *B*, representing the bias stability of the sensors.

In Table 2, the corresponding values are summarized. The power spectral densities (PSDs) of the corresponding noise components are shown. These values can be used directly in the filters for GNSS/IMU processing [22].

Some sensor manufacturers provide not only numerical noise characteristics but also ADEV plots. However, for low-cost sensors, such as those used in smartphones, manufacturers do not provide such information at all. For the sensors under consideration, only Epson EG320N installed in NovAtel PwrPak7 is accompanied by a datasheet with information on noise values. For the other sensors in this study, the manufacturers do not provide graphs or values for the noise components of interest.

Therefore, programs were written in the Python language to perform OADEV analysis and display the results. For this purpose, the allantools [23] and matplotlib [24] libraries were used. Also, the pandas library [25] was used to read the measurement data from text files.

The pseudocode to perform the task is given in Algorithm 1.

Proprietary software rokubun_core version 75.3.1 (developed at the Rokubun company Barcelona, Spain) was used for the GNSS/IMU combined solution.
**Algorithm 1** Compute and plot OADEV for sensor data1:Initialize sensor_files with paths to sensor output files2:**for** each file in sensor_files **do**3:      data ← read_sensor_data(file)4:      **for** axis in {x, y, z} for both gyroscope and accelerometer **do**5:            axis_data ← extract_data_for_axis(data, axis)6:            oadev_result ← compute_oadev(axis_data)7:            [N, B] ← find_noise_values(oadev_result)8:            plot_oadev(oadev_result)9:            output_values(N, B)10:     **end for**11:**end for**12:**function** read_sensor_data(file)13:     **return** data from file14:**end function**15:**function** extract_data_for_axis(data, axis)16:     **return** data corresponding to specified axis17:**end function**18:**function** compute_oadev(axis_data)19:     **return** OADEV computation result20:**end function**21:**function** plot_oadev(oadev_result)22:     Plot the OADEV result23:**end function**24:**function** find_noise_values(oadev_result)25:     Analyze the OADEV plot to find N and B values26:     **return** [N, B]27:**end function**28:**function** output_values(N, B)29:     Display or save the N and B values30:**end function**

## 4. Results

This section analyzes the results of the OADEV applied to the collected data from the three IMUs considered. Then, the results of the combined GNSS/IMU processing for the NovAtel PwrPak7 and Samsung Galaxy Note10 devices are presented.

### 4.1. EPSON G320N OADEV Results

**Figure 3:**
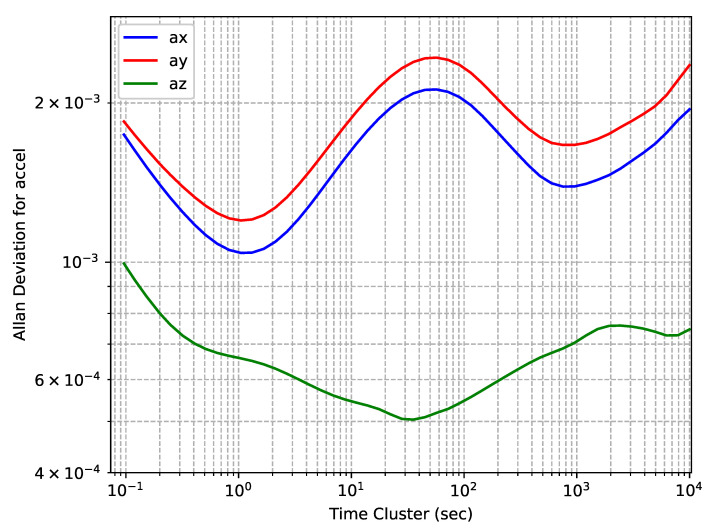
Epson EG320N accelerometers’ OADEV curves.

The accelerometers’ OADEV plots for EPSON G320N embedded in a NovAtel PwrPak7 receiver are shown in Figure 3. One can observe that the shape of the curves for the *x*- and *y*-axis sensors are not as expected, there is a noticeable oscillation in both. Similar peculiarities have been found in other similar studies [26], which are probably explained by the specifics of sensor manufacturing. On the other hand, the difference observed in the *z*-axis sensor, which was directed vertically, with regard to the other two axes suggests that there is a relationship with position in the obtained results due to correlation with the gravity vector. To verify the relationship between this feature and the direction of free-fall acceleration, an additional experiment was conducted to collect data with a change in the device orientation, so that the *y*-axis was directed vertically. The shapes of the obtained curves did not differ from those in the original experiment.

**Figure 4:**
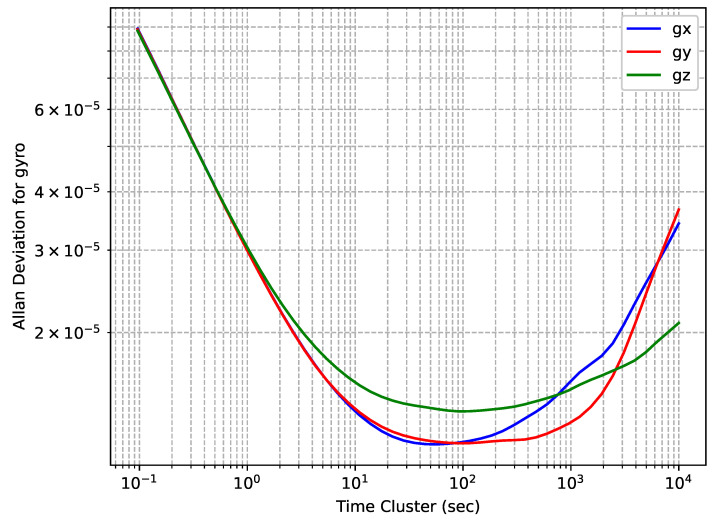
Epson EG320N gyroscopes’ OADEV curves.

The gyroscopes’ OADEV plots are shown in Figure 4. One can see that the curves are very close, although there is a slight difference for the sensor on the *z*-axis.

The sensor manufacturer Epson provides not only numerical noise data but also OADEV plots for several sensors. However, no such information is provided for this particular Epson EG320N sensor made specifically for NovAtel. The values for bias instabilities and velocity and angular RW that were obtained after the OADEV analysis are in a good agreement with the data from the datasheet provided by the manufacturer. The values are close to each other and to the reference values at the level of a fraction of an order of magnitude. In Table 3, the computed values are shown along with reference values from the datasheet in the last column of the table.

### 4.2. STM LSM6DSR OADEV Results

**Figure 5:**
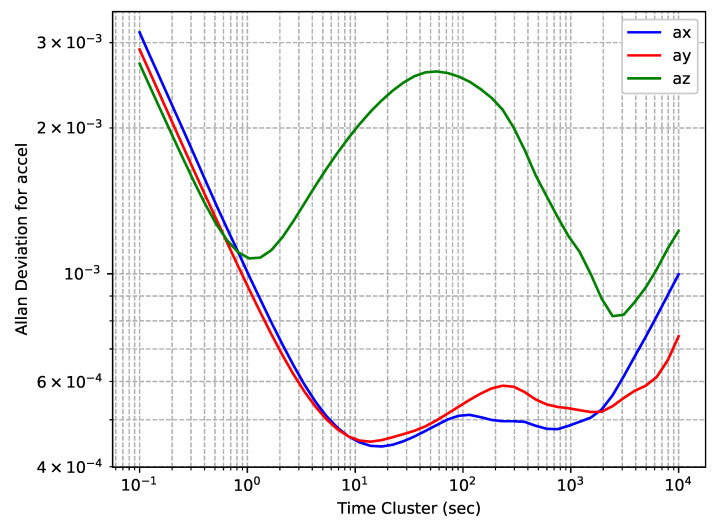
STMicroelectronics LSM6DSR accelerometers’ OADEV.

OADEV plots for the accelerometers of the STM LSM6DSR IMU embedded in a Samsung Galaxy Note10 Smartphone are shown in Figure 5. Here, the bending on the accelerometer of the *z*-axis is observed. This axis was directed vertically. When changing the position of the phone, the same pattern was observed for other axes directed vertically; though, this does not affect the values of the calculated noise components.

**Figure 6:**
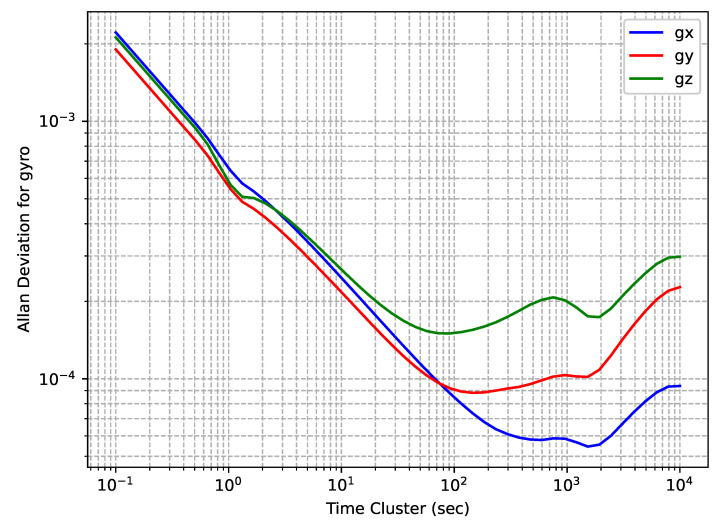
STMicroelectronics LSM6DSR gyroscopes’ OADEV.

Figure 6 shows the OADEV plots derived for the gyroscopes.

The resulting noise components are presented in Table 4. There are no reference values from the manufacturer, since it does not provide such information. It can be seen that the values for velocity RW for the accelerometers and angular random walk for the gyroscopes, respectively, are similar for sensors of all axes, but there are significant differences in biases. Apparently, this is due to the low grade of the IMU, since no such differences are observed for the IMU of higher grade in the NovAtel PwrPak7 receiver.

### 4.3. TDK MPU9250 OADEV Results

**Figure 7:**
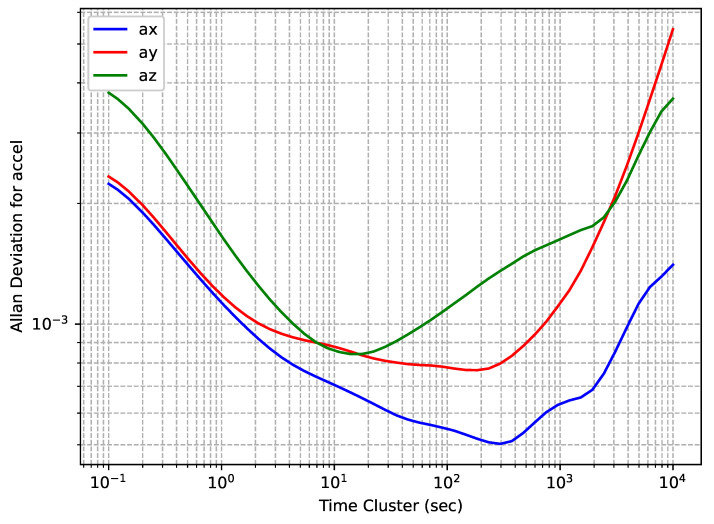
InvenSense TDK MPU9250 accelerometers’ OADEV.

**Figure 8:**
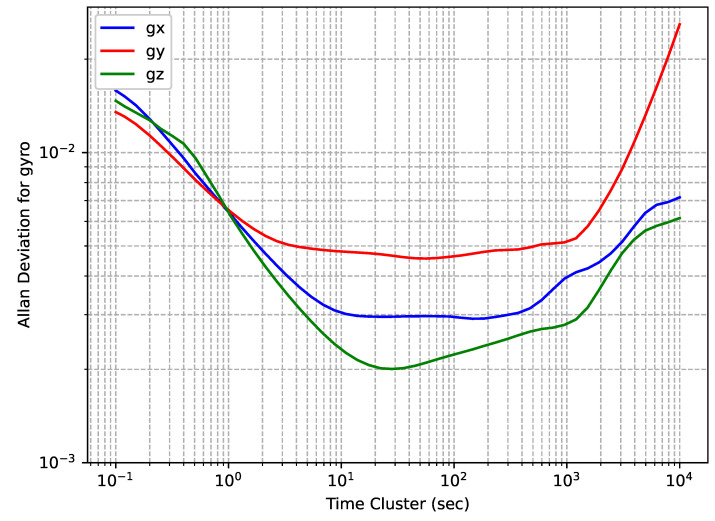
InvenSense TDK MPU9250 gyroscopes’ OADEV.

OADEV plots for the accelerometers of the TDK MPU9250 IMU embedded in a Rokubun Argonaut receiver are shown in Figure 7, while OADEV plots for the gyroscopes are shown in Figure 8.

The IMU manufactured by TDK has the lowest grade among the tested ones, and it can be seen that the curves on different axes show significant differences among them in a wide range of time cluster values. This is also reflected in the calculated values of noise components that are shown in Table 5. As with the STM LSM6DSR, for this IMU, the manufacturer does not provide information on the noise components of interest in the datasheet, and therefore, Table 5 lacks a column for the reference value.

### 4.4. Combined Solution Results

In order to assess the calculated noise parameters, combined processing of GNSS/IMU measurements in a loosely coupled (LC) mode was performed. This is one of the common techniques for navigation using satellite and inertial measurements and is described in detail in ref. [22]. The Rokubun software rokubun_core version 75.3.1 implements such processing using an extended Kalman filter (EKF) in Cartesian Earth-centered, Earth-fixed frame (ECEF). The EKF was chosen due to the absence of extreme non-linear dynamics in typical navigation tasks, which aligns well with EKF’s linearization approach. The EKF’s ease of implementation, high performance in mildly non-linear systems, and the intuitive clarity in filter tuning—directly relating to the modeling of sensor noise components—make it a popular choice for GNSS/IMU combined navigation [27]. The LC technique involves using the solution obtained from GNSS-only processing—positions, velocities and receiver clock biases (so-called PVT)—which forms the timescale to which the measurements are tied. Those positions and velocities are pseudo-measurements and are used to estimate the state parameters (attitude, position, and velocity errors and sensor biases). A system state vector on each estimation step is the following:

(2) $$x_{LC}=\left(\begin{array}{c} \delta \psi \\ \delta v\\ \delta r\\ b_{a}\\ b_{g} \end{array}\right),$$

where δψ is a vector of errors of three attitude angles —Euler angles—which determine the body-to-ECEF transform matrix, δv is the velocity error vector, δr is the position error vector, $b_{a}$ is a vector of accelerometer biases, and $b_{g}$ is a vector of gyroscope biases. Thus, the total number of parameters to be estimated is 15.

**Figure 9:**
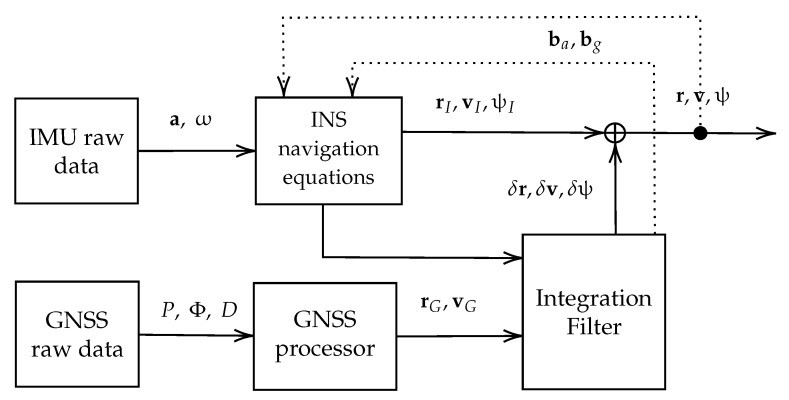
Loosely coupled processing flow chart.

The LC-processing flow chart is shown in Figure 9. The IMU provides measurements—3D acceleration vector a and angular rate ω. The INS navigational equations are the mechanization equation for calculating positions and velocities based on IMU measurements and IMU corrections (biases). The raw IMU data provide three-dimensional acceleration a and angular rate ω vectors. From these data, the INS navigation equation is used to calculate the position $r_{I}$, velocity $v_{I}$, and orientation of the device, ψ. In turn, GNSS measurements, code *P*, and phase Φ pseudoranges and Doppler (*D*) are processed in the GNSS processor and provide position and velocity ($r_{G}$ and $v_{G}$, respectively). These data are used in the integration filter as pseudo-observations. The estimation in the filter results in corrections to the INS-computed position, velocity, and orientation angles (δr, δv, and δψ respectively) and sensor biases, $b_{a}$ for accelerometer and $b_{g}$ for gyroscope. These biases are used in subsequent INS calculations until the next estimation.

There are certain features in the Rokubun software’s implementation that are worth mentioning, in particular, with each IMU data sample EKF’s state estimation error covariance matrix is updated, even if there is no GNSS PVT solution and no parameter estimation is performed. In this case, the propagation of the solution errors and the continuity of the filtering process are ensured after the appearance of GNSS following a period of absence of measurements.

The formula to update the state estimation error covariance matrix P is the following:

(3) $$P^{+}=\Phi \left(P^{-}+\frac{1}{2}Q\right)\Phi^{T}+\frac{1}{2}Q,$$

where a superscript ‘+’ denotes an updated P and superscript ‘−’ an old matrix from a previous step, Φ is a transition matrix, and Q is a system noise covariance matrix:

(4) $$Q=\mathrm{diag}\left(S_{grn},S_{arn},S_{gb},S_{ab}\right),$$

where $S_{grn}$ and $S_{arn}$ refer to the power spectral densities (PSDs) of the gyroscope and accelerometer random noises, and $S_{gb}$ and $S_{ab}$ are, respectively, the gyroscope and accelerometer bias variation PSDs. These PSD values are derived from the noise components that are computed from the OADEV analysis and are described in Table 2.

The dataset of GNSS and IMU measurements collected using the NovAtel PwrPak7 in an automotive scenario was used for processing. The drive duration was 14 min, the GNSS measurement rate was 1 Hz, and the IMU rate was 125 Hz. A GNSS signal was available throughout 84% of the route. To assess the combined solution and effect of noise parameters, the computed trajectory was compared with the reference trajectory, a high-precision post-processing solution obtained using the NovAtel Inertial Explorer software version 9.00 [28]. To calculate it, GNSS and IMU were combined in tightly coupled mode, with multi-path forward and backward processing. In addition, for the GNSS part, the precise point positioning (PPP) method [29] was used.

To perform the calculations in LC mode using the Rokubun software, the values obtained in Inertial Explorer for the epochs corresponding to the GNSS observations were used as positions and velocities. The idea of this processing mode is to minimize the impacts of the GNSS solution’s noise on the computed parameters, in order to isolate the effect of the IMU’s noise parameters as much as possible.

**Figure 10:**
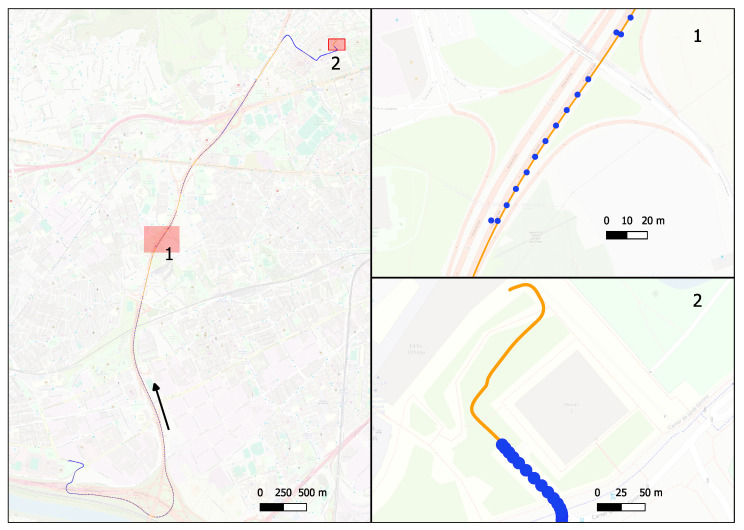
NovAtel PwrPak7 trajectory. Blue dots are GNSS points used for the LC combined solution, and orange dots are INS parts of the trajectory that correspond to GPS outages. The arrow indicates the route direction. The two regions labeled 1 and 2 and highlighted in red on the left are shown zoomed on the right. The first of the segments, with missing GNSS solutions, is shown at the top, the segment with the final part of the route is shown at the bottom.

**Figure 11:**
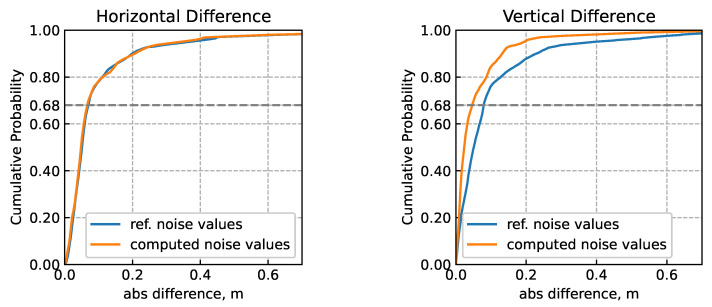
Cumulative distribution function of horizontal (**left** plot) and vertical (**right** plot) differences in solutions with respect to the reference trajectory.

Two trajectories were calculated, one using the noise values calculated from the OADEV analysis, the other using the values given in the manufacturer’s specifications. The corresponding route followed for data collection and LC-solution is illustrated in Figure 10. Both trajectories are very close, although a slight improvement can be seen when using the calculated values. The comparison of the two trajectories is presented in Table 6 with statistical characteristics of the horizontal, vertical and 3D differences between the calculated and reference trajectories. In Figure 11, the cumulative distribution functions for vertical and horizontal differences with the reference trajectory are shown. It can be seen that the difference is very small for the vertical component, but a definite decrease in errors in the horizontal plane can be observed. This improvement, although not very significant, appears to be due to a more accurate detection of sensor errors thanks to a more adequate tuning of the navigation filter.

**Figure 12:**
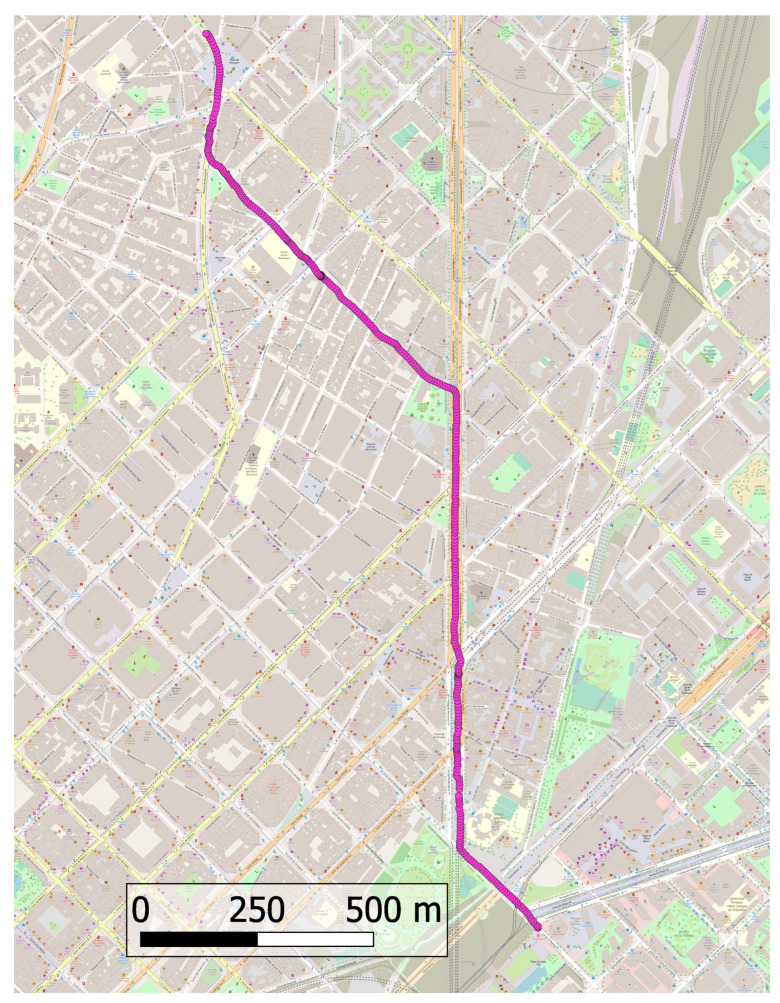
Samsung Galaxy Note10 loosely coupled trajectory calculated with obtained noise values.

**Figure 13:**
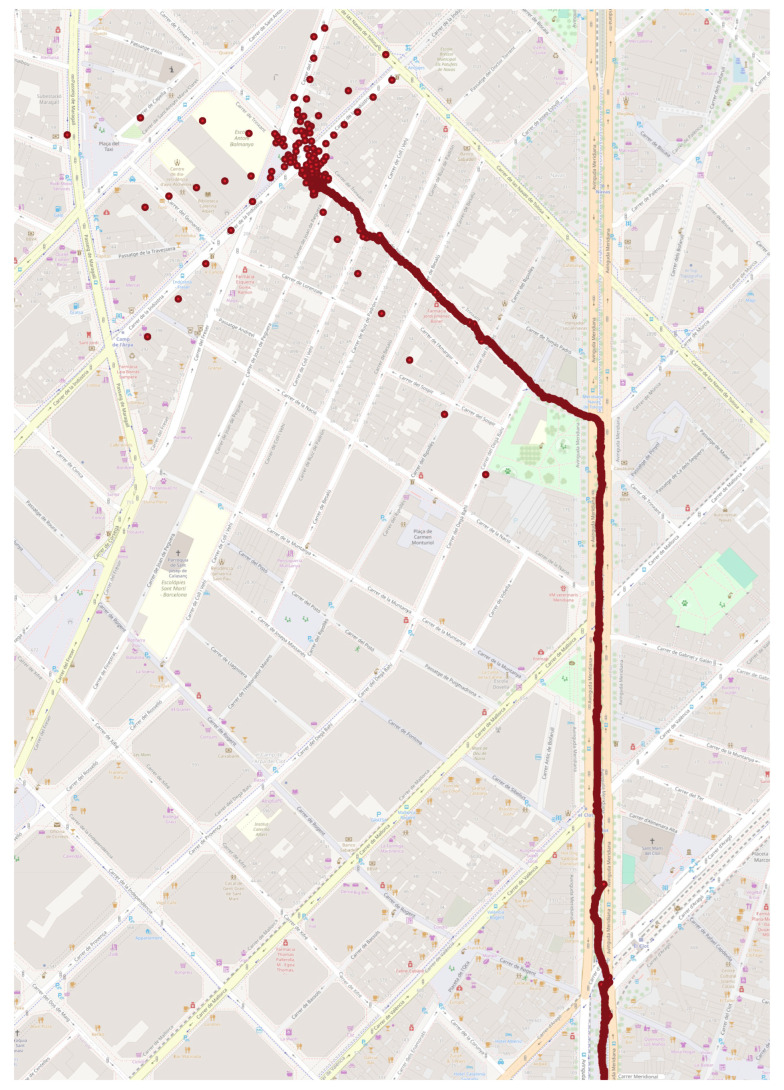
Samsung Galaxy Note10 loosely coupled trajectory with changed noise values leading to a divergent solution.

A similar analysis was not performed for the other two devices due to not being able to obtain a highly accurate reference trajectory. The GNSS and IMU measurements for this specific experiment were collected in an urban environment with the device mounted on the handlebar of a bicycle. The GNSS solution used in the loosely coupled processing was calculated with the RTKLIB version demo5 b34c software [30] in post-process kinematic mode. The solution was obtained with the calculated noise values and the resulting trajectory does not show any divergence, i.e., it does not show unrealistic deviations and no numerical singularities in the calculations were found; Figure 12 shows this trajectory. Another solution was obtained with all the noise values increased by one order of magnitude. The resulting trajectory is shown in Figure 13. In this case, there were more deviations along the trajectory, and also at a certain point the solution diverged, which is clearly visible in the upper part.

**Figure 14:**
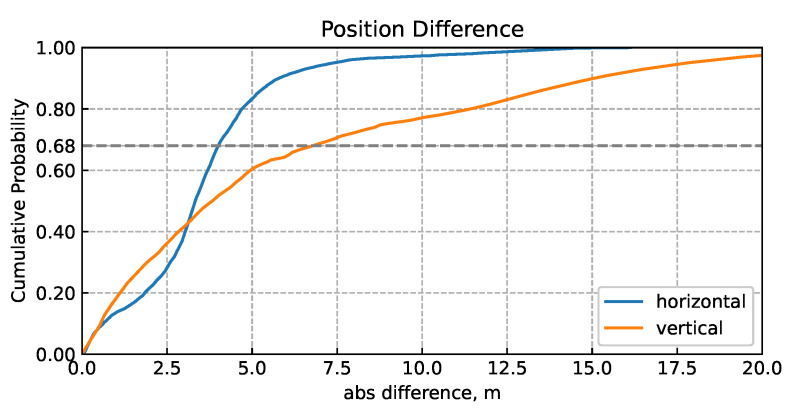
Cumulative distribution function of horizontal (blue line) and vertical (orange line) differences between two LC solutions for Samsung Galaxy Note10.

Since there was no reference trajectory for this case, a qualitative assessment based on the comparison between them was made, assuming that the first trajectory was more correct. Also, for the comparison, the final part of the second trajectory was discarded, where the solution clearly diverges, producing outliers of hundreds of meters. The statistical results of the comparison are summarized in Table 7, and a CDF plot of the differences is also given in Figure 14. Even in the part of the trajectory with the non-divergent solution with incorrect noise parameters, significant differences between the trajectories, reaching tens of meters, are observed.

## 5. Conclusions

In this work we collected data and evaluated the noise components of three different-grade sensors using OADEV. They showed a close match between the noise characteristics of the Epson G320 sensor and the data provided by the manufacturer, and validated the results using combined GNSS/IMU processing. Thus, we propose a structured approach, which is of interest to those who work with IMU sensors of different levels and use them for combined navigation tasks. It does not require complex laboratory facilities and equipment or sophisticated processing methods and, consequently, it can be easily reproduced.

The results obtained support the finding that the noise parameters of sensors working in static mode can be used for the combined GNSS/IMU processing in dynamic mode. The values for the only IMU tested for which the manufacturer provides noise values, the IMU Epson EG320N, were close to the manufacturers’ specifications. The obtained values are not necessarily optimal for GNSS/IMU processing. In different usage scenarios they can be modified, e.g., to incorporate the effect of external vibrations, but they can serve as the values to start with.

In general, the noise estimation procedure can be carried out according to the proposed methodology for any sensors before using them for combined GNSS/IMU processing, but the present contribution mostly addresses the situation where electronic component manufacturers do not provide the required specifications in the datasheet, as for the STMicroelectronics LSM6DSR and InvenSense TDK MPU9250.

The methodology presented in this paper can also be applied in case the values are present in the IMU datasheet. Usually, noise values are given for a specific frequency, but the operating frequency of the IMU may be chosen differently by the manufacturer of the device in which the IMU is integrated. In such a case, the noise values may differ from the reference values and they could be derived from an OADEV analysis. 

## Figures and Tables

**Table 1:** Key sensor specifications.

Parameter	EPSON G320N	STM LSM6DSR	TDK MPU9250
3-axis accelerometer	✓	✓	✓
3-axis gyroscope	✓	✓	✓
3-axis magnetometer	×	✓	✓
Measurement data rate ^1^, Hz	125	100	100
Accelerometer range ^2^, g	±5	±8	±4
Accelerometer resolution, m/s^2^	2.99 × 10^−8^	0.002	0.0012
Gyroscope range, °/s	±150	±1000	±500
Gyroscope resolution, °/s	1.22 × 10^−7^	0.035	0.01523

^1^ Rate is based on output time-stamps; ^2^ values are from the current device manufacturer settings.

**Table 2:** Noise components in IMU.

Noise Description	Allan Variance	PSD
Random walk ^1^, *N*	$\frac{N^{2}}{\tau }$	$N^{2}$
Bias (in)stability ^2^, *B*	$\frac{2B^{2}\ln2}{\pi }$	$\frac{B^{2}}{2\pi f}$

^1^ For accelerometer *N* is a velocity random walk (RW) in $m/\sqrt{s^{3}}$ for gyroscope *N* is an angular RW $\mathrm{rad}/\sqrt{s}$. ^2^ For accelerometer *B* is in $m/s^{2}$, for gyroscope *B* is in rad/s.

**Table 3:** Epson EG320N OADEV results. The calculated noise components are shown, the last column gives the values from the manufacturer’s documentation.

Noise Component	*x*-Axis	*y*-Axis	*z*-Axis	Ref. Value
Velocity RW, m/s/$\sqrt{s}$	5.40 × 10^−4^	5.72 × 10^−4^	3.08 × 10^−4^	8.33 × 10^−4^
Acc. bias instability, m/s^2^	5.57 × 10^−9^	8.01 × 10^−9^	1.53 × 10^−9^	1.22 × 10^−9^
Angular RW, rad/$\sqrt{s}$	2.81 × 10^−5^	2.80 × 10^−5^	2.78 × 10^−5^	2.91 × 10^−5^
Gyro bias instability, rad/s	1.74 × 10^−5^	1.75 × 10^−5^	2.04 × 10^−5^	1.70 × 10^−5^

**Table 4:** STMicroelectronics LSM6DSR OADEV results.

Noise Component	*x*-Axis	*y*-Axis	*z*-Axis
Velocity RW, m/s/$\sqrt{s}$	9.99 × 10^−4^	9.20 × 10^−4^	8.58 × 10^−4^
Acc. bias instability, m/s^2^	6.63 × 10^−4^	6.79 × 10^−4^	1.62 × 10^−3^
Angular RW, rad/$\sqrt{s}$	7.00 × 10^−4^	6.01 × 10^−4^	6.70 × 10^−4^
Gyro bias instability, rad/s	8.84 × 10^−5^	1.54 × 10^−4^	2.26 × 10^−4^

**Table 5:** InvenSense TDK MPU9250 OADEV results.

Noise Component	*x*-Axis	*y*-Axis	*z*-Axis
Velocity RW, m/s/$\sqrt{s}$	9.61 × 10^−4^	9.62 × 10^−4^	1.60 × 10^−3^
Acc. bias instability, m/s^2^	7.64 × 10^−4^	1.16 × 10^−3^	1.27 × 10^−3^
Angular RW, rad/$\sqrt{s}$	6.07 × 10^−3^	5.64 × 10^−3^	6.25 × 10^−3^
Gyro bias instability, rad/s	4.45 × 10^−3^	6.87 × 10^−3^	3.04 × 10^−3^

**Table 6:** Statistics of the differences between the calculated trajectory and the reference trajectory. Mean, standard deviation (std. dev.) and maximum 3D differences are given in meters.

	Horizontal		Vertical		3D		
	**Mean**	**Std. dev.**	**Mean**	**Std. dev.**	**Mean**	**Std. dev.**	**Maximum**
Datasheet values	0.10	0.16	0.10	0.14	0.15	0.20	1.84
Computed values	0.09	0.16	0.06	0.09	0.12	0.17	1.76

**Table 7:** Statistics of the differences between the trajectory obtained with computed noise values and the trajectory with erroneous noise values.

	Mean, m	Std. Dev., m	Maximum, m
Horizontal diff.	3.53	2.32	16.12
Vertical diff.	5.97	5.80	31.51
3D diff.	7.30	5.81	32.49

## Data Availability

The data collected for this work are available on reasonable request.

## Abbreviations

The following abbreviations are used in this manuscript:

ADEV	Allan Deviation
CSV	Comma-separated values format
GNSS	Global Navigation Satellite System
ECEF	Earth-centered, Earth-fixed frame
EKF	Extended Kalman filter
IMU	Inertial measurement unit
INS	Inertial navigation system
LC	Loosely coupled
MEMS	Micro-electromechanical system
OADEV	Overlapped ADEV
PPP	Precise point positioning
PSD	Power spectral density
Std. dev.	Standard deviation
PVT	Position, velocity, and time as result of GNSS solution
RW	Random walk
